# Supplementary material for: Enduring Effects of the COVID-19 Pandemic on Food Access, Nutrition, and Well-Being in Rural Appalachia
Source: Int J Environ Res Public Health. 2024 May 4;21(5):594. doi: 10.3390/ijerph21050594 (PMC11120804; doi:10.3390/ijerph21050594)
Supplement: Supplementary file 1 [file ijerph-21-00594-s001.zip › ijerph-2877006-supplementary.pdf]

University of Kentucky Family and Consumer Sciences Extension
Project Title: Healthy Eating and Active Living to Reduce Rural Obesity through Extension
Target audiences: *health coalition members, community residents*
Instrument Title: Focus Group Questions

We are asking you to take part in a 60-minute focus group about resources available for making healthy choices in your community, the need for additional resources to promote healthy choices, and the barriers and facilitating factors to support healthy eating habits and physical activity in your community. You do not have to respond if there are any questions you are uncomfortable answering. You will be compensated for your time at the end of today's focus group: all participants will receive a $40 gift card. If you agree to participate, we invite you to sign our consent form at this time.

Introduction Script:

Good morning / afternoon. Thank you for taking the time to be here today. My name is ____________ and I will be leading this focus group. The notetakers and I are from the University of Kentucky Cooperative Extension Service. Your participation is greatly appreciated, and we hope to have a good discussion this morning / afternoon. Please silence your cell phones so we may have fewer distractions during our time together.

My role is to ask questions and keep the conversation moving. We want to create a safe place for everyone to share their opinion, so please be respectful and let people finish their thoughts before responding. We encourage you to share your perspective, even if it is different from others. There are no right or wrong answers to the questions asked, only different points of view. You are free to participate as much or as little as you feel comfortable. We have one note taker sitting in the back of the room and will also be tape recording this focus group session.

We are here today to talk about food choices and physical activity. We will also talk about how the COVID-19 pandemic, higher food costs, and supply chain issues have impacted your ability to access or have enough food for you and your family. We would like to identify or better understand any facilitating factors and barriers to making healthy choices in your community. The responses you share today will help us identify needs for future programs in the community.

Let's begin by going around the room. Please introduce yourself by first name only.

End Script:

Thank you very much for your time. It is important for us to understand the available resources for healthy choices within a community, as well as what needs to be developed. Your answers are important and useful to us. We appreciate your time and thank you again for attending and sharing your opinions.

Target audiences: *broad representation of community residents*

Questions:

"To get started, tell me about your community."

- Probe: "Tell us what is good/positive about your community."
    a. Probe if needed: "What are some of your favorite things about your community?"
- Probe: "What are some challenges that your community faces?"
    a. Probe if needed: "What are some of the hardest things you think your community faces and deals with?"
- Probe: "What do you see as opportunities in your community?"
    a. If needed: "Such as places, programs, or even people that may be helpful to you or others in your community. Why is that?"

"The next set of questions we are going to ask are related to food choices. These questions will be related to resources in your community, the need for additional resources, and any challenges you may have to make healthy choices in your community. We are going to start by having everyone go around and share the one word that comes to mind when they think of food in this community."

1. "How do people in your community get food?"
    - Probe: "Where do people shop for food most often?"
    - Probe: "What other locations in the community are people getting food from?"
        - i.e., food drops, community garden, food pantries, schools, etc.
    - Probe: "Are you aware of the community garden located at Turkey Creek Church of the Nazarene?"
2. "What are common foods and meals eaten in your community?"
    - Probe: "How often are fruits and vegetables eaten?"

"Some of the questions we have for you today are related to the coronavirus pandemic – COVID-19 – and how it has impacted you, your family, and your community. Where would you say your community is with COVID-19?"

- Probe: "Does COVID-19 continue to impact day-to-day life in your community?"
- Probe: "What changes from COVID-19 are lasting in your community?"

1. "Can you talk about how COVID-19 impacted how people get food in your community?"
    - (e.g., Are people gardening/farming more? Food pantries? Churches?)"
2. "How many people in your community do you think ran out or were worried about running out of food because of the coronavirus pandemic in the last year?"

"We now want to ask a few questions related to purchasing food in your community, particularly over the last year."

1. "What changes have you seen in the last year related to the cost of food?"
2. "Why do you think food prices have changed in the last year?"
3. "How have price changes impacted how you get food?"
    - Probe: "Has this influenced how much food you get? If so, how?"

- Probe: “Has this influenced the type of food you get? How?”
- Probe: “Has this impacted the quality of food you eat? How?”

4. “What foods have been the most difficult to get in your community?”
    a. Probe: “What are people in your community doing to get these foods?”

“We are now going to transition to a few questions related to physical activity. For purposes of our discussion, physical activity can include swimming, biking, running, but it also can include activities that cause a small increase in your breathing and heartrate such as brisk walking or carrying a light load for 10 minutes. . Like our previous questions, we are going to start by having everyone go around and share the one word that comes to mind when they think of being active in this community.”

1. “How have physical activity levels changed in your community over the last year?”
    - Probe: “Have you noticed others in your family or your neighborhood changing their exercise patterns? This could be walking, spending more time outside, gardening, etc.”
2. “What resources are available in your community that allow you to walk for physical activity?”
    - Probe: “Has anyone seen or heard of the new Story Walk?”
    - Probe: “How often do people go to local parks (i.e., Tomahawk, Warfield, Grassy, Pigeon Roost, etc.)?
    - Probe: “Do you use them, why or why not?”
3. “Have you noticed improvements to local parks and trails in your community?”
    - Probe: “Do you think people are using them more often because of the improvements?”

“Before we wrap up, we’d like to know – where do you get information from in your community?”

- I.e., news, health information, information about community events, etc.

1. “Why do you refer to these resources specifically?” Probe: “Is it easiest, you think it’s the most trustworthy, do they talk about things that interest you.”

“Thank you for your thoughtful responses so far. We want to end our time together by asking a couple of summary questions.”

1. “Over the last few years, there have been a number of challenges which impact daily life (i.e., COVID-19, flooding, supply chain issues, etc.), what has had the greatest impact in this community?”
    - Probe: What challenge has had the biggest impact on how you eat?
    - Probe: What challenge has had the greatest impact on being active?
2. “Last, we want to know what do you want to see for the future of this community?”

Thank you so much for your time and participation today.
